# The Science of Animal Behavior and Welfare: Challenges, Opportunities, and Global Perspective

**DOI:** 10.3389/fvets.2015.00016

**Published:** 2015-05-28

**Authors:** Jeremy N. Marchant-Forde

**Affiliations:** ^1^United States Department of Agriculture – Agricultural Research Service, Livestock Behavior Research Unit, West Lafayette, IN, USA

**Keywords:** animal welfare, behavior, ethology, emotions, welfare assessment, ethics

Animal welfare science is a relatively new scientific discipline, evolving mostly from within veterinary medicine over the latter half of the twentieth century into an independent specialty in its own right. Originally, the field of study was heavily focused on animal behavior (ethology), but it has emerged into a truly multi- and inter-disciplinary science, encompassing such sciences as behavior, physiology, pathology, health, immunology, endocrinology, and neuroscience, and influenced by personal and societal ethics. The first academic organization devoted to the scientific study of animal welfare was established in 1966 as the society for veterinary ethology (SVE), demonstrating its veterinary roots by being then affiliated with the British Veterinary Association. The world’s first Professor of Animal Welfare was appointed 20 years later at the University of Cambridge’s Department of Clinical Veterinary Medicine, and in 1991, the SVE became the International Society for Applied Ethology, in recognition of its geographical spread and its evolution from veterinary medicine. Over the last quarter of a century, there has been further expansion of the field and now animal welfare science is represented in many universities’ veterinary medicine and animal science departments across the world. Animal welfare science has become part of the core curriculum for many veterinary degrees, is a recognized specialty qualification within the veterinary professions of Europe, USA, and Australia and courses in animal welfare science as a stand-alone discipline are offered worldwide at Bachelor’s, Master’s, and Doctorate degree levels. Within research, there have been similar patterns of expansion and a spread from a heavy focus on farm animal welfare to the welfare of zoo, laboratory, and companion animals and the impacts of humans on wildlife. There continue to be studies that compare the welfare of populations within systems, but there is also more attention given to gaining in-depth knowledge of the welfare of individual animals, knowing that populations are not homogenous and that individuals within the same system may be experiencing quite different welfare states. We not only continue to use “traditional” welfare indicators but also work to develop novel indicators for use in experimental settings or in the field. As our fundamental knowledge base increases, we look for increasing application and we respond to challenges that arise from our own research questions and findings and from societal needs. In this paper, I will focus on a number of the areas that I see represent Grand Challenges within our discipline.

## Animal Welfare Science in Focus

What I hope to address under this title are the challenges that define how the science of animal welfare may progress over the next few years. I will present my views of the challenges and opportunities that welfare assessment presents and how we adapt and adopt methodologies within animal welfare science to obtain greater understanding, quantification, and qualification of an animal’s welfare state. Historically, animal welfare has been defined under one of three over-arching, and intersecting, themes or approaches. These are biological functioning, “naturalness,” and feelings. The biological functioning theme of animal welfare enables us to focus on discreet measurable parameters, such as health indicators, production measures, measures of physiological functioning, and incidence of behaviors, and combine multiple measures to draw an overall picture of the welfare of the given animal at the time, or prior to, when the measures are taken. The “naturalness” theme focuses on the extent to which the animal is leading, or can lead, a life in which it is free to express its natural behavioral repertoire, with the idea that an animal being able to experience or fulfill its inherent nature, will have good welfare. The third theme concerns the feelings, emotions, or affective states of the animal, with the broad idea that for an animal to be experiencing good welfare, it should not only be devoid of negative emotions, such as anxiety or fear, but should also be experiencing positive emotions, such as pleasure or happiness. As I said above, these themes or approaches do not each exist in isolation and it is commonly acknowledged that there is a degree of overlap between them, and that in attempting to best establish the welfare state of an individual, there should be elements drawn from all three approaches (1).

### Animal welfare and emotional states

**Figure 1:**
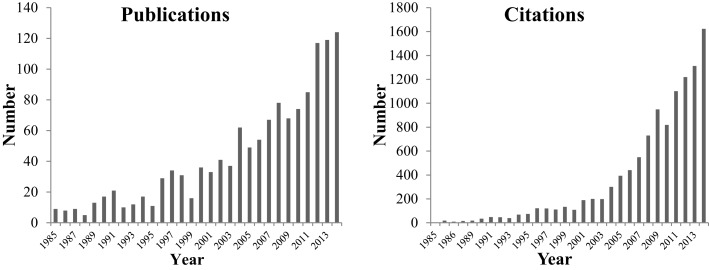
**Number of publications and citations of studies that include animal welfare and emotions over the time period 1985–2014 (Web of Science)**.

The emotional states of animals in our care have been increasingly important in research terms, over the last few years. A Web of Science search combining the terms “animal welfare” with a number of different terms of emotionality shows a rapid increase in both publications and citations in the last 10 years (Figure 1). Out of a total of nearly 1350 publications, 130 focus on positive emotional states, whereas over 1200 address negative emotional states and the most cited paper (which includes open commentary from 45 other academics) concerns animal suffering (2).

However, the more recent trend is an increasing interest in positive rather than negative emotional states and a recent review article on measuring positive emotions in animals in relation to welfare is topping the table in terms of citations per year (3). In terms of recent experimental work, the most influential studies have been those using cognitive bias to assess emotional state (4, 5). Other work in this area has included the use of functional near infrared spectroscopy to assess changes in cortical perfusion and neuronal activity in the study of mood and emotional reactions (6). There is no doubt that further adaptation of methods used within cognitive science and behavioral neuroscience will yield new fundamental insight into animal emotions, and thus its welfare. An example could be further development of electroencephalogram (EEG), which although currently used in sleep (7) and euthanasia/slaughter studies (8), may also yield insight into emotional state (9).

### Animal welfare and quality of life

A more recent term and concept that has been used within the companion animal welfare field is that of quality of life (QoL). The term “Quality of Life” originated within the human sociological/geographical/medical fields in 1950s and 1960s and within the human field, depending on context, is taken to encompass such measures as wealth, physical and social environment, health, and biological functioning. Critically, within the human medical field, it also incorporates the psychological well-being of the individual and health-related QoL (HRQoL) is defined as being “subjective and multidimensional, encompassing physical and occupational function, psychological state, social interaction, and somatic sensation” (10) and invariably includes self-reporting. Certainly, the emphasis of human QoL is on affective states in general and positive affective states, in particular. From an animal perspective, this of course presents problems. QoL for animals has been defined as encompassing animal welfare and the subjective feelings of the animal regarding its life, but that it can only be inferred from behavioral, physiological, and other measures (11). So it would seem that for many animal welfare scientists, there is a large degree of synonymy between their own working definition of “animal welfare” and that of “animal QoL,” but there is also some confusion (12).

Although there has been recent use and assessment of QoL in animals – mostly companion animals – there is certainly a degree of resistance within the broader animal welfare field to use of the term, either because of the perceived time-span limitations of its coverage or because of the anthropomorphism and subjectivity associated with a definition that includes assessment of an animal’s psychological state by indirect methods. However, there is increasing research focus within our field on indicators of positive welfare, rather than negative welfare, and on measures of the animal’s affective state, meaning that as our methods and measures evolve and refine, we may see a shift toward a concept of animal welfare that is more in line with definitions of QoL than our current definitions of animal welfare.

### Animal welfare assessment in the field

Increasing our fundamental knowledge about an animal’s welfare is part of our remit as animal welfare scientists. We also have to seek to apply that new-found knowledge to improve the welfare of animals under our care (13). When assessing animal welfare within an experimental setting, there is greater focus on the individual animal and more options in terms of the parameters that can be measured. Out in the “field,” be it within a farm, zoo, or lab animal facility or with companion animals in homes, stables, or shelters, there are many more limitations on the types of data that can be collected and where large facility populations are concerned, there will be focus on the group rather than on individuals within the group.

The reasons for assessing welfare in the field will differ depending on the setting and the species. For farm animals, ensuring a certain welfare standard may be tied to improving productivity and also to marketing of the end product, based on consumer or retailer demand (14), as seen by the many auditing or quality assurance schemes that exist for farm animal production. For laboratory animals, there is again a societal demand for a minimum welfare standard to be met, and there is sound argument that testing is carried out on animals devoid of poor welfare and altered biological function (15). For zoo animals, it may be relevant again to the “consumer” – i.e., the zoo-going public – and enforced by legislation (16), but also to better serve the biological needs of the animals to facilitate reproduction in those species subject to conservation efforts and because it is an ethical obligation. The Association of Zoos and Aquariums has a stated policy that “Animal and human health, safety, and welfare are never compromised” (17). Welfare assessment among companion animals is less developed and is more focused on environments where populations are being housed – e.g., humane shelters – rather than home settings with few individuals. Within shelters, there is an increasing understanding that physical and social environments that improve welfare also improve long-term adoptability (18).

The majority of field welfare assessment protocols for farm animals are heavily weighted in terms of environmental assessment – that is they gather much information on such things as the physical housing, the management techniques, the health and production records, and relatively less information on measures that are taken on the animals themselves. On large farms, there is a trade-off between time and the number of animals that can be assessed, and thus assessment has to be carried out on a representative sample, that will give an indication of the overall, or “population average” welfare rather than the welfare of individuals within the population. Also, the animal-based measures that are taken are often more concerned with direct measures of health and disease, or indirect measures of behavior, such as skin lesions, rather than the behavior itself. There has been some good progress in farm animal assessment recently (19), but more animal-centered methods and, in particular, methods that give quantifiable insight into the psychological lives of animals are needed, together with the development and validation of new indicators, such as tear-staining (20, 21) that are relatively easily discernable to the observer, that may enable more objective assessment of individuals within a given population. For laboratory animals, even in a large facility, there should be greater attention to the individual than seen with on-farm assessment (22). Welfare assessment should comprise components that describe or quantify physical, physiological/biochemical, and psychological states, and may include scoring scales for such things as body weight, coat condition, respiration rates, ocular discharge, feces condition, and provoked behavioral response. With well-defined scoring scales, the overall assessment of an individual’s welfare can be objectively quantified and intervention carried out if a threshold total score is reached (22). Zoo animals are mostly housed in limited numbers and welfare assessment at an individual level is commonplace. Zoo keepers often have many years of experience with particular species and individual animals and are thus well-placed to assess welfare through sometimes quite subtle changes in appearance or activity, which can be objectively recorded and reported over time, using available tools such as WelfareTrack^®^ (23). Superimposed upon individual-level assessment, there are also increasing numbers of projects within the zoo community that seek to combine multi-institutional data and information to identify patterns in welfare issues for particular species, that may be previously unidentified without such collaboration (24).

## Animal Welfare and the “Big Picture”

Animal welfare is an important societal concern and as a scientific field of study, animal welfare is one of the branches of specialized science that is most accessible and inherently interesting to the majority of the general public. This is a good thing, as public importance and relevance opens minds and doors to expansion and application of our science. As I touched on above, the original concept of QoL from a human point of view was to encompass aspects of the physical and social environment. For a great many people, their relationships or interactions with animals constitutes a large part of their daily life, and the quality of that life, be it with animals owned as companions or as a source of income, or with free-living animals within their ecosystem. As the global population continues to grow, there are a number of societal “Big Picture” challenges that are being, and will continue to need to be addressed, and with which animal welfare is intrinsically tied. Thus, as animal welfare scientists, it is more essential than ever that we do not live wholly within the bubble of animal welfare science and that we expand our horizons outside a relatively narrow scientific discipline to interact with, inform and learn from others working on global issues, which themselves are interconnected.

### Animal welfare, population growth, global food security, and sustainability

Food security is access by all people at all times to enough food for an active, healthy life (25) and it means having a reliable source of food and sufficient resources to purchase it. A family is considered food secure when its members do not live in hunger or fear of starvation. For those of us working in animal agriculture, we are constantly reminded of projected global population growth over the next 35 years, with an expected increase in global population from the current 7.2 billion up to 9.6 billion by 2050. Concomitant with this increase in overall population is a projected increase in overall food demand and a projected per capita increase in demand for food from animal sources, especially in developing countries. At present, more than 1 billion people are food insecure. As the global population increases, the number of people with an insecure food supply will also dramatically increase unless there is an increase in food production, and improvements in food distribution and storage. Given the sizeable stress that this demand for animal and crop production will place on planetary resources, it is not surprising that “sustainability” has become a keyword within agriculture as a whole. The main emphasis is on economic and environmental or ecological sustainability, but there is also the critical element of ethical or social sustainability (26). The projected increase in animal production will be achieved by increases in animal numbers and further increases in productivity – output per unit input. However, although current projections do show an increase in animal production, it is also probable that the actual increase will be buffered by a shift in diet away from animal products to foods from crop sources, either through concerns about animal welfare or sustainability/land-use efficiency or through perceived health benefits associated with a vegetarian/vegan diet. Twenty-five years ago, reasons for not eating animal products were mostly due to animal welfare (66%) and health reasons (26%) rather than environmental/ecological reasons (1%) (27). Five years ago, a much larger survey found that reasons for dietary animal-product avoidance were now shifted toward health (40.1%) and environmental/ecological (38.1%) reasons and away from animal welfare reasons (16.5%) (28). Although these studies are not directly comparable, they do highlight that consumers are also looking more at the “big picture” and identifying the relationship between animal production and sustainability.

Although over the last few decades, livestock farming in developed countries has been characterized by decreasing farm numbers, increasing farm size, and increased intensification, its ethical acceptability has been increasingly questioned. There will still be increasing intensification in newly industrialized and developing countries to meet the growing food demand, albeit not completely unquestioned. Fully industrialized economies, however, will continue to see mounting vociferous opposition to farming systems that do not meet society’s demand for production that is ecologically, environmentally, and ethically acceptable. Within the ethical acceptability boundary is the notion that animal farming systems must meet or exceed standards of animal welfare deemed acceptable by the given society, and this should not be overlooked (29). Even though a system may be environmentally and economically sustainable, if the animals kept within it are subject to housing conditions or production demands deemed unacceptable in terms of animal welfare, then consumer acceptance will evaporate, demand for the product will decrease and the system of production will ultimately become unsustainable – as seen in confinement housing systems such as veal crates, battery cages for laying hens, and gestation crates for sows. Alternatively, public antipathy may reach such a level that rather than a gradual decline in consumer demand forcing change, legislation may be enacted that outlaws the production system, either within a localized legislative framework (e.g., single state or country) or a more broader one (e.g., federal body). Examples of such housing systems include battery cages for laying hens, crates for veal calves, and crates for gestating sows.

However, it is not only housing systems being questioned. The public is also concerned with the animal’s own biological functioning and whether continued “improvements” in parameters such as litter size, growth rates, egg or milk production can be supported if the animal’s welfare is negatively impacted in combination. Another animal welfare concern is the potential impact of increased food production on wildlife, with decreasing natural habitats as land is converted to crop or animal farming and the culling of predators or wildlife that may consume crops or compete with farmed animals for resources. The issue of animal welfare as a component of sustainability and food security is important as we implement or adapt current systems for use in developing countries, and design new systems for use in a dynamic, economically interconnected world. Although the integration of an animal welfare emphasis into livestock and crop production systems in developing countries can also result in higher biodiversity, restoring habitat, reversing the impact of traditional production systems (30), and improving QoL, the standards of acceptable animal welfare are greatly changed by the level of food security and those of us living in food secure households must be aware that our own baseline of acceptability may be quite different from those struggling to feed themselves regularly.

Finally, another often overlooked aspect of growth in global population and wealth is a concomitant increase in the global population of companion animals, particularly in those countries with developing economies. For example, India has seen a 90% increase in cat and dog population between 2002 and 2012 (31), and even the U.S. cat and dog population has increased 15% in that time. In terms of big picture implications, this rapidly expanding population also puts further pressure on food supply and hence food security and sustainability. Increasing feral populations of companion animal species can also impact wildlife and animal and human health.

### Animal welfare and climate change

Scientific consensus is that the world’s climate is undergoing change (32). Temperatures and sea levels are rising and extreme climatic events are increasing in number. Much of this is attributed to an increase in greenhouse gases as a result of human activities, including some from animal production (33). Climate change will impact wildlife welfare in many ways, such as affecting habitat and food sources, decreasing water availability and shifts in ranges of disease vectors (34) to the extent that many species will ultimately be at threat of extinction (35). Companion and zoo animals may likewise be subjected to vector-borne diseases in new geographical areas and challenged by changes in thermal environment. Climate change will also present potential challenges in crop and animal production at a time when, as noted above, overall demand will be increasing. The projected further changes over the twenty-first century are variable, depending on the projection model used, but the general implications include further increases, and perhaps fluctuations in temperature and greater variability in precipitation, resulting in reduced or modified availability of water for agricultural purposes.

So far, there have been very few studies that have tried to quantify the impact of real-life climate change over the last few decades on livestock production, and those that have been carried out are focused on modeling projected impacts of future climate change, or models of disease transmission given the increased range of disease vectors. However, under experimental conditions, it is well known that heat and cold have very obvious effects on productivity, and indeed on welfare. Heat stress reduces appetite, reduces growth, affects reproduction, decreases milk and egg production and, at critical levels, can lead to heat stroke and death (36). Shifts in both maximum and minimum temperatures may result in more animals being exposed to both heat and cold stress events, thereby impacting their welfare.

Changing precipitation patterns may result in current pastoral-based systems having to adapt either in response to moving away from drought-susceptible areas or having to surrender high-quality pastureland over to crop production, and moving to reduced quality areas, with breeds of livestock that are not so well suited for the new, harsher environment. The changes in climate may also impact disease transmission with disease vectors, such as insects, becoming established in previously unrecorded areas.

### Animal welfare, animal health, and food safety

A recent study estimated that each year in the U.S., about 3% of the population suffers from an illness due to 1 of 31 known foodborne pathogens. According to these data, there are approximately 9.4 million illnesses, of which 56,000 require hospitalization, resulting in 1350 deaths (37). The U.S. Centers for Disease Control and Prevention estimate much higher figures. They not only include the above data set but also include data due to “unspecified agents” causing the symptoms of acute gastroenteritis. Combined, there are 48 million illnesses, 128,000 hospitalizations, and 3000 deaths (38). These are not all animal-product related, and further estimates within the U.S. put animal-related foodborne illness as a cause of 42% of illnesses, 46% of hospitalizations, and 43% of deaths, with major illness-causing agents identified as *Listeria*, *Salmonella*, *Campylobacter* spp., and *Norovirus* (39). Expand these estimates to a global population and the impact of unsafe food, especially in areas with limited access to medical treatment is great. The World Health Organization estimate 1.9 million children die each year from diarrheal illnesses and there is a major WHO initiative underway to estimate the “global burden of foodborne diseases,” which will report in 2015.

How does food safety fit with animal welfare? There is plenty of evidence of a direct link between animal welfare and animal health. Animals under stress are often immune-compromised and are more susceptible to disease. Higher levels of disease within an animal population can result in an increased risk of bacterial or viral contamination within food products and there is also a risk of medication residues in food products if withdrawal protocols are not stringently followed. Although the public often consider extensive production systems as being high welfare and high health, the reality is that decreased biosecurity in extensive systems may, in fact, increase exposure to, and incidence of certain diseases (40). There remains a greater need to consider the “farm-to-fork” chain as a whole and not focus on individual stages of the chain in isolation. Only with a foundation of high welfare/low stress/high health production systems can we begin to make real advances in the safety of the final products. Again, this “big picture” theme is intrinsically linked to the others.

## Conclusion

Originally, animal welfare science was heavily behavior-based. As a scientific discipline in its own right, animal behavior has perhaps been unfairly thought of as a “soft” science and is still subject to misapplication by non-ethologists assuming they can study behavior without adequate expertise or training. Done right, behavior is valid and precise but it has taken the integration of perceived “harder” sciences such as physiology, immunology, and pathology for animal welfare science to evolve independence and specialty status. There is also the irony that as we now strive to develop and incorporate measures that will identify and quantify emotional state, we are perhaps returning to our ethological roots and becoming more reliant on behavior as a key component of our science.

As I hope I have illustrated above, there are real opportunities for animal welfare science to continue to expand our fundamental knowledge of many facets of biological functioning of the animals with which we interact, and enable us to apply our new-found knowledge to continue to improve our animals’ lives. A major advantage for our discipline is its relative accessibility to the general public and to scientists working in other fields. Animal welfare science is therefore positioned to play a role in some of the big issues confronting our global society and the opportunities afforded by inter-disciplinary collaboration should be embraced.

## Conflict of Interest Statement

The author declares that the research was conducted in the absence of any commercial or financial relationships that could be construed as a potential conflict of interest.
